# Editorial: Clinical prospective of SGLT2 inhibitors in atherosclerosis

**DOI:** 10.3389/fcvm.2022.1040649

**Published:** 2022-11-11

**Authors:** Annalisa Capuano, Emilio Clementi, Giuseppe Paolisso

**Affiliations:** ^1^Department of Experimental Medicine, University of Campania “Luigi Vanvitelli”, Naples, Italy; ^2^Department of Biomedical and Clinical Sciences, University of Milan, Milan, Italy; ^3^Department of Advanced Medical and Clinical Science, University of Campania “Luigi Vanvitelli”, Naples, Italy; ^4^Mediterranea Cardiocentro, Naples, Italy

**Keywords:** atherosclerosis, SGLT2 inhibitors, cardiovascular diseases, acute myocardial infarction, heart failure, peripheral artery diseases

## Introduction

SGLT2 inhibitors have recently come to prominence in research due to their anti-atherosclerotic effect. This collection of articles highlights that these effects can be expressed both at the cardiovascular and cerebrovascular levels with a reduction in the risk of such events (1). Also of significant interest are the data showing that SGLT2 inhibitors can have an anti-arrhythmic effect, a finding that confirms previous *in vivo* experimental evidence in animals (2). The well-known beneficial effect of SGLT2 inhibitor administration on renal function could explain the highlighted data of a lower incidence of contrast medium side effects during revascularization procedures in acute myocardial infarction (AMI) patients (3). The molecular mechanisms related to the anti-atherosclerotic effect of SGLT2 are very complex and can range from molecular to clinical effects.

## Molecular mechanisms for atherosclerosis

Atherosclerosis is a vascular disorder characterized by lipid particles, immune cells, and an extracellular matrix complex, which allows the development of plaques in the arterial wall. Such plaques may undergo erosion or rupture, being the cause of several cardiovascular diseases (CVD), encompassing AMI, stroke, heart failure (HF), and peripheral arterial disease. Genetic factors and continuous exposition to oxidative stress are additional factors that could be taken into account to explain the genesis and development of atherosclerosis. Furthermore, modifiable risk factors such as obesity, diabetes mellitus, dyslipidemia, cigarette smoking, physical inactivity, and hypertension contribute further (4).

Once the initial lesions occur, the first stage comprises the endothelial dysfunction associated with disturbed shear stress, combined with a chronic inflammation state, changes in endothelial permeability, and infiltration of monocytes in the intimal layer. Oxidized-LDL can be responsible for damage-associated molecular patterns secretion that initializes an innate immune response by Toll-like receptors (TLR). As a result, an immune response develops, since vascular cell adhesion molecule 1 (VCAM-1) is expressed by endothelial cells, which recall other immunogenetic cells such as monocytes and leukocytes (4, 5). Monocytes transform themselves into activated M1 and M2 macrophages. The balance between the pro-inflammatory capacity of M1 and the anti-inflammatory propriety of M2 might be responsible for inflammation's occurrence or disappearance (4). A unique role also seems to be played by the lipids whose accumulation is associated with the appearance of foam cells and activation of nucleotide-binding domain-like receptor protein 3 (NLPR3) inflammasome complex, which *per se* promotes the release of pro-inflammatory cytokines, contributing to the development of atherosclerotic plaque (5–7).

Oxidative stress and cellular senescence may contribute further to endothelial dysfunction and atherosclerosis. Reactive oxygen species (ROS) prompt the synthesis of pro-inflammatory cytokines, thus stimulating the expression of adhesion molecules, allowing monocytes to transmigrate into the vessel wall. On the other hand, ROS can favor scavenger receptors' expression on vascular smooth muscle cells, which is responsible for lipid accumulation and switch in foam cells (4).

At the end of the atherosclerotic process, we can distinguish between two different plaques: i) stable plaques made up of a thick fibrous cap, which can regress with an appropriate lifestyle and/or drug therapy or may occlude the vessel lumen; ii) vulnerable or unstable plaques very rich in macrophages made up by a thick lipid core and a thin fibrous cap, which very often proceeds toward rupture and complication such as AMI and stroke (8–10).

## Which role for SGLT2 inhibitors on cardiovascular diseases?

SGLT2 inhibitors display advantageous cardiac effects independent of the well-known anti-hyperglycemic effect. In particular, SGLT2 inhibitors reduce the occurrence of heart failure with reduced ejection fraction (HFrEF). This phenomenon is independent of glucose-metabolic control and attributable to both indirect and direct cardiac effects (9, 10). Recent studies have highlighted that SGLT2 inhibitors exerted their cardiovascular protective activity through hemodynamic effects (mainly related to an increased diuresis/ natriuresis and decline in arterial blood pressure) and metabolic impact (9–12).

## Do SGLT2 inhibitors play a role as anti-atherosclerotic drugs?

The potential anti-atherosclerotic role of SGLT2 inhibitors might be due to multifactorial effects related to a downgrade of inflammatory molecule secretion, reduction of macrophage infiltration, improvement in autophagy impairment, and opposition to endothelial dysfunction. To date, we can distinguish between glucose-dependent and independent anti-atherosclerotic effects. SGLT2 inhibitors, by decreasing plasma glucose levels, may decline macrophage inflammatory response since macrophages preferentially utilize glucose as an energy source. Such a hypothesis is strengthened by a positive linear correlation between neutrophil plasma levels measured at 24 h from AMI and admission plasma glucose levels or plasma troponin levels in AMI type 2 diabetic patients treated by SGLT2 inhibitors (13). Such data supported the hypothesis that such an anti-inflammatory effect might be attributed to tighter metabolic control of stress hyperglycemia independently of glucose control measured by HbA1c levels. As far as the glucose-independent anti-atherosclerotic process is concerned, recent studies have shown that empagliflozin might inhibit *per se* the nucleotide-binding domain-like receptor protein 3 inflammasome (NLRP3). Furthermore, dapagliflozin reduced the maturation and secretion of inflammatory markers (13), an effect associated with ketone inhibition of the NLRP3 (4). Indeed, SGLT2 inhibitors increase plasma beta-hydroxybutyrate with a parallel decline in fasting plasma insulin levels and a consequent rise in insulin sensitivity (13), all such activities correlated to the inhibition in NLRP3.

## Clinical evidence for the anti-atherosclerotic role of SGLT2 inhibitors

Several recent studies have highlighted the anti-atherosclerotic role of SGLT2 inhibitors. In particular, the Real Catalonia study evidenced in heart failure patients with type 2 diabetes indicated that canagliflozin administration significantly lowered inflammatory biomarkers after 3, 6, and 12 months (14). In addition, the DEFENSE study demonstrated that dapagliflozin improved endothelial dysfunction in patients affected by type 2 diabetes mellitus (15). The sub-analyses of the CVD-REAL and CVD-REAL2 studies highlighted a reduced risk of AMI associated with SGLT2 inhibitors (16). As recently reported, three further studies (EMMY, DAPA-MI, and EMPACT-MI) confirm these data (16–19).

## Conclusions

SGLT2 inhibitors may play an anti-atherosclerotic role through a well-defined anti-inflammatory role, which could be related to prevention in endothelial dysfunction and a reduced risk of AMI in type 2 diabetic patients.

## Author contributions

All authors listed have made a substantial, direct, and intellectual contribution to the work and approved it for publication.

## Funding

This work was funded by PON Ricerca e Innovazione 2014 – 2020 ARS01_01270.

## Conflict of interest

The authors declare that the research was conducted in the absence of any commercial or financial relationships that could be construed as a potential conflict of interest.

## Publisher's note

All claims expressed in this article are solely those of the authors and do not necessarily represent those of their affiliated organizations, or those of the publisher, the editors and the reviewers. Any product that may be evaluated in this article, or claim that may be made by its manufacturer, is not guaranteed or endorsed by the publisher.
